# A successful preterm vaccination program in a neonatal unit in a developing country

**DOI:** 10.1016/j.heliyon.2019.e02857

**Published:** 2019-11-30

**Authors:** Lloyd Tooke, Byron Louw

**Affiliations:** Neonatal Department, Groote Schuur Hospital, University of Cape Town

**Keywords:** Public health, Infectious disease, Immune system, Vaccines, Pediatrics, Preterm, Paediatrics, Vaccines

## Abstract

**Background:**

Although preterm infants are at a greater risk from vaccine preventable diseases, there are frequent delays in vaccine administration with great variability between units. There is little data from developing countries. Groote Schuur Hospital in Cape Town, South Africa starting vaccinating preterm infants from 2014.

**Objectives:**

To determine whether vaccines were given at the correct chronological age and whether there were side effects or logistical problems.

**Methods:**

For a six month period, all infants who were still admitted at 6 weeks of age were included. Date of vaccination and side effects were recorded.

**Results:**

60 infants were included. 57 (95%) received their 6 week vaccines. 68% received the vaccines on time, 10% early and 17% late. Reasons for delay included oxygen dependence and concerns about sepsis. There were no side effects.

**Conclusions:**

It is possible to implement a successful vaccination program for preterm infants in a low resourced setting.

## 1 Introduction

Benjamin Franklin is quoted as saying ‘An ounce of prevention is worth a pound of cure’. This is certainly true for preterm infants who are at greater risk from vaccine preventable diseases than their term counterparts [1, 2]. This is due to both humoral and cellular immunological factors as well as decreased passive maternal antibody transfer which predominantly occurs after 34 weeks gestation [3]. Bronchopulmonary dysplasia (BPD), a complication of prematurity which is exacerbated by ventilation and oxygen toxicity increases the risks of pulmonary infection further [4]

Vaccination is the most effective means of preventing infections and should be administered at chronological, rather than corrected age. Although preterm infants do not mount the same antibody response as their term counterparts, protective levels are obtained [5, 6]. Lack of awareness of this recommendation, as well as concerns about safety, result in frequent delays of vaccination with considerable variation between units [7, 8, 9]. Most reports are from units in developed countries.

Groote Schuur Hospital (GSH) neonatal unit is one of 2 government tertiary referral centres for the Western Cape province in South Africa. GSH admits over 500 VLBW infants per year, with approximately 40% of these delivered due to complications of severe pre-eclampsia/hypertension [10]. Up until 2014, no vaccines, except polio and BCG, were administrated in the unit. All subsequent vaccines (see Table 1) were administered at community clinics following discharge from hospital, usually starting at the corrected age of 6 weeks.

**Table 1:** Vaccination schedule for South Africa until 6 months of age.

Age of child	Vaccines Needed
At birth	OPV(0); BCG
6 weeks	DTaP-IPV-Hib-HBV(1); PCV(1); RV(1); OPV(1)
10 weeks	DTaP-IPV-Hib-HBV(2)
14 weeks	DTaP-IPV-Hib-HBV(3); PCV(2); RV(2)

OPV: Oral Polio Vaccine; BCG: Bacille Calmette Guerin; DTaP-IPV-Hib-HBV: Diphtheria, Tetanus, acellular Pertussis - Inactivated Polio vaccine - H.Influenza type b combined; PCV: Pneumococcal Conjugated vaccine; RV: Rotavirus vaccine.

In July 2014, after reviewing the literature, it became practice to implement routine vaccination at chronological age for all infants still admitted in our neonatal unit at 6, 10 and 14 weeks of age. Pharmacy, medical and nursing staff were updated on the policy change. In the event of medical concerns regarding the suitability of vaccination for an infant, then the attending neonatologist was to be consulted.

Following implementation of this new policy, a study was undertaken to evaluate whether the new protocol was effective and safe.

## 2 Objectives

1) To determine whether vaccines were given at the correct chronological age.
2) To describe any administrative/logistical problems
3) To record any side effects of the vaccinations

## 3 Methods

Data were collected for 6 months from October 2014 until April 2015. All infants who were still admitted at 6 weeks of age were included in the study. Data collected included gestational age, birthweight, age (days) at which vaccines were received, problems with administration, and any adverse events up to 72 h post immunisation. Data were obtained from folders and medication charts. To ensure all eligible infants were included, patient lists were cross-checked with the GSH Vermont Oxford Network (VON) database, an international database which collects information on all infants ≤1500g. All data were entered onto Excel spreadsheets.

The study was approved by the Human Research Ethics Committee of the Health Sciences Faculty of the University of Cape Town.

## 4 Results

A total of 60 infants were still hospitalised at 6 weeks of age. Median birth weight was 920g and median gestation age 28 weeks (IQR 27–29 weeks). Fifty-seven received their 6 week vaccines and 52 infants were discharged before they were 10 weeks of age. Of the five remaining infants, all received their 10 week vaccinations and were discharged before 14 weeks of life.

Three infants did not receive their 6 week vaccines. Two of the infants were deemed too unwell (necrotising enterocolitis and heart failure due to an inoperable lesion). Both infants subsequently died (on day 47 and day 76 of life). For the third infant, it can be assumed that the vaccination opportunity was missed as there was no record of the vaccine nor reasons why the vaccine should have been omitted.

**Figure 1:**
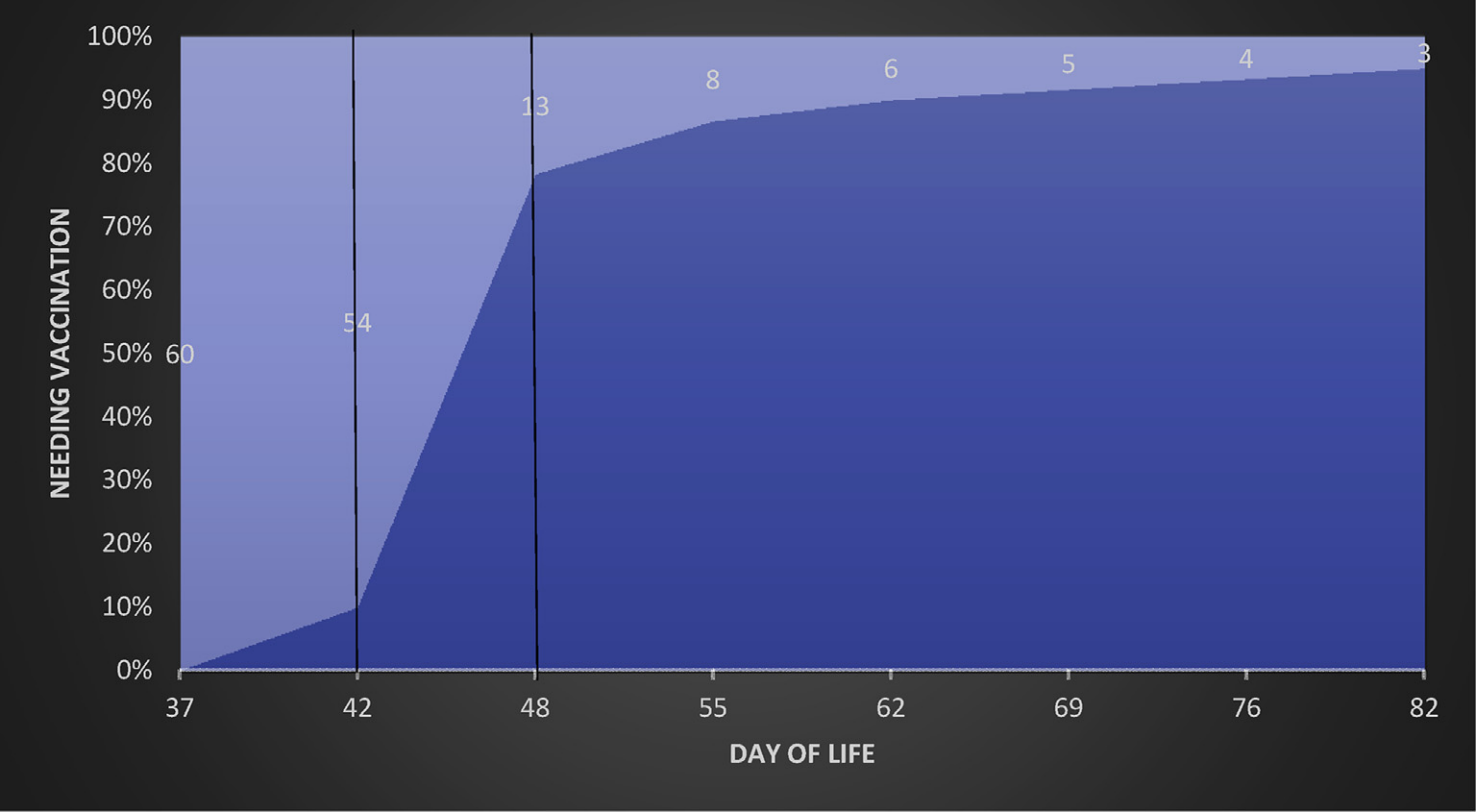
Cumulative 6 week vaccine administration.

Of the 60 infants, 41 (68%) received 6 week immunizations correctly between day 42 and day 48. Six infants received vaccines early (up to 5 days) and 10 were late (after day 48) (Figure 1). Reasons for late administration included: oxygen dependency (4 infants), unknown (3 infants), concerns of sepsis (2 infants) and post-surgical procedure (1 infant).

Of the 10 week vaccinations, 2 were given at the correct time and 3 were delayed (2 of these were as a result of late 6 week vaccinations).

There were no reports of any side-effects including fever, skin reactions or apnoeas in any of the infants within 72 h of vaccination. There were no cases of rotavirus diarrhoea in any vaccinated or unvaccinated infant.

There were few logistical problems. Despite initial concerns from some nursing staff regarding the administration of intramuscular injections into premature limbs, most were amenable to administering the vaccinations with no extra training required. During the 6 months of the study, there were seven occasions that either DTaP-IPV-Hib-HBV (Pentaxim) or the hepatitis B vaccine was not available due to stock shortages. These however, were quickly replenished.

## 5 Discussion

It is possible to effectively introduce an immunization program for preterm infants in a developing country. It is essential that there is buy-in and capacity from both pharmacy and the nursing staff. In our unit, we achieved a 95% vaccination rate, with 68% of these correctly administered during their 7^th^ week of life. Two infants were deemed too unwell to receive vaccines. Five infants received vaccines early (a maximum of 5 days) which was unlikely to affect their efficacy. Of the 10 infants who received late vaccinations, many of these were unnecessarily delayed. Three infants had no apparent reason for delay and Montague et al showed that respiratory decompensation after vaccination was rare, even amongst infants with BPD and advised that BPD or oxygen dependence should not be a reason to delay vaccines [11].

It was reassuring that no side effects were reported. Apnoea has previously been associated with the use of vaccines in preterm infants [12], but this was thought to be associated with the whole cell pertussis vaccine [13]. South Africa changed to the acellular pertussis vaccine in 2009, which has been shown to not increase apnoeic events compared to control [14].

Subsequent to this study, vaccine use has remained at high levels in our unit, with few contra-indications for administration at the chronological age. It would be important to continue measuring the efficacy of the policy to ensure sustained compliance [15].

## 6 Conclusion

It is possible to implement and sustain a preterm vaccination program in low resourced settings where the benefit of chronological vaccination may be highest. Other neonatal units in developing countries, who are not yet vaccinating appropriately, should investigate implementing similar guidelines and thereby also “provide ounces of prevention to prevent pounds of cure”.

## Declarations

### Author contribution statement

L. Tooke: Conceived and designed the experiments; Analyzed and interpreted the data; Contributed reagents, materials, analysis tools or data; Wrote the paper.

B. Louw: Performed the experiments; Analyzed and interpreted the data; Contributed reagents, materials, analysis tools or data.

### Funding statement

This research did not receive any specific grant from funding agencies in the public, commercial, or not-for-profit sectors.

### Competing interest statement

The authors declare no conflict of interest.

### Additional information

No additional information is available for this paper.
